# Proteasome Particle-Rich Structures Are Widely Present in Human Epithelial Neoplasms: Correlative Light, Confocal and Electron Microscopy Study

**DOI:** 10.1371/journal.pone.0021317

**Published:** 2011-06-17

**Authors:** Vittorio Necchi, Patrizia Sommi, Alessandro Vanoli, Rachele Manca, Vittorio Ricci, Enrico Solcia

**Affiliations:** 1 Department of Human Pathology and Genetics, University of Pavia, Pavia, Italy; 2 Centro Grandi Strumenti, University of Pavia, Pavia, Italy; 3 Pathologic Anatomy Service, Fondazione IRCCS Policlinico San Matteo, Pavia, Italy; 4 Department of Physiology, University of Pavia, Pavia, Italy; Uppsala University, Sweden

## Abstract

A novel cytoplasmic structure has been recently characterized by confocal and electron microscopy in *H. pylori*-infected human gastric epithelium, as an accumulation of barrel-like proteasome reactive particles colocalized with polyubiquitinated proteins, *H. pylori* toxins and the NOD1 receptor. This proteasome particle-rich cytoplasmic structure (PaCS), a sort of focal proteasome hyperplasia, was also detected in dysplastic cells and was found to be enriched in SHP2 and ERK proteins, known to play a role in *H. pylori*-mediated gastric carcinogenesis. However, no information is available on its occurrence in neoplastic growths. In this study, surgical specimens of gastric cancer and various other human epithelial neoplasms have been investigated for PaCSs by light, confocal and electron microscopy including correlative confocal and electron microscopy (CCEM). PaCSs were detected in gastric cohesive, pulmonary large cell and bronchioloalveolar, thyroid papillary, parotid gland, hepatocellular, ovarian serous papillary, uterine cervix and colon adenocarcinomas, as well as in pancreatic serous microcystic adenoma. *H. pylori* bodies, their virulence factors (VacA, CagA, urease, and outer membrane proteins) and the NOD1 bacterial proteoglycan receptor were selectively concentrated inside gastric cancer PaCSs, but not in PaCSs from other neoplasms which did, however, retain proteasome and polyubiquitinated proteins reactivity. No evidence of actual microbial infection was obtained in most PaCS-positive neoplasms, except for *H. pylori* in gastric cancer and capsulated bacteria in a colon cancer case. Particle lysis and loss of proteasome distinctive immunoreactivities were seen in some tumour cell PaCSs, possibly ending in sequestosomes or autophagic bodies. It is concluded that PaCSs are widely represented in human neoplasms and that both non-infectious and infectious factors activating the ubiquitin-proteasome system are likely to be involved in their origin. PaCS detection might help clarify the role of the ubiquitin-proteasome system in carcinogenesis.

## Introduction

By means of electron and confocal microscopy of *H. pylori*-infected gastric epithelium, we recently described a novel cytoplasmic structure characterized by the accumulation of particles resembling in size, shape and inner structure, the barrel-like proteasome machinery and showing immunoreactivity for several proteasome component proteins [1]. This proteasome particle-rich cytoplasmic structure (PaCS) was also found to selectively concentrate polyubiquitinated proteins, NOD1, the intracellular *H. pylori* receptor [2], several bacterial products, including VacA and CagA toxins as well as SHP2 and ERK proteins, known to interact with CagA in gastric carcinogenesis [1], [3]. In addition to foveolar-superficial epithelium, PaCSs were also found inside gastric dysplastic lesions and in cultured neoplastic cell lines of gastric and non-gastric origin, especially when incubated with *H. pylori* or its virulence factors [1].

These findings, together with mounting evidence supporting a role of proteasome in cancer development and progression [4], prompted us to investigate human epithelial neoplasms for the presence of PaCS. Various solid neoplasms of different origin, histology, genetic background and malignancy grade were investigated for PaCS by ordinary or correlative light, confocal and electron microscopy after immunostaining for proteasome components and polyubiquitinated proteins. PaCSs were detected in gastric, lung, thyroid, parotid, hepatocellular, colon, ovarian and uterine cervix cancers as well as in pancreatic serous microcystic adenoma.

## Materials and Methods

Both formalin-fixed paraffin embedded surgical specimens and formaldehyde–glutaraldehyde fixed, osmium post-fixed, Epon-Araldite embedded specimens from the same gastric, pancreatic, liver, colon, lung, parotid gland, thyroid, breast, ovary and uterine cervix neoplasms, undergone surgery in the years 1984–1990, were retrieved from the files of the Department of Pathology, University of Pavia and Policlinico San Matteo Hospital. A total of 45 epithelial neoplasms were obtained. For comparison, we also investigated archival resin (either Epon-Araldite or hydrophilic LWR) blocks from gastric endoscopic biopsies, fixed in a formaldehyde-glutaraldehyde solution with or without osmium post-fixation [1]. No specimen specifically and/or exclusively devoted to the present study was taken. The study has been approved by the Ethics Committee of Fondazione IRCCS Policlinico San Matteo (Pavia, Italy) as a reinvestigation of archival material along the same line (i.e., diagnosis of pathology in neoplastic patients) as for the original written consensus, without the need of further consent and with communication to the national “Garante” of the privacy.

Paraffin sections were stained with hematoxylin-eosin, Giemsa or immunoperoxidase [5] using antibodies directed against proteasome 20S, αβ subunits, or the 19S, S2 subunit (both rabbit polyclonal antibodies, Calbiochem, La Jolla, CA), polyubiquitinated proteins (mouse monoclonal, FK1 clone, Enzo Life Sciences International, Plymouth Meeting, PA), and the sequestosome marker p62 protein (also known as SQSTM1) (rabbit polyclonal, H-290, Santa Cruz Biotechnology, Santa Cruz, CA). Semithin (about 0.5 µm) sections from Epon-Araldite blocks were either stained with toluidine blue in a pH 8 borax solution for conventional light microscopy or processed for immunofluorescence with 20S proteasome (rabbit polyclonal, PW8155, Enzo), ubiquitinated protein (mouse monoclonal, FK2 clone, Enzo) or p62 protein (Santa Cruz) antibodies followed by Alexa 488-labelled anti-mouse or anti-rabbit-IgG, as appropriate. A TCS SP2 confocal laser scanning microscope (Leica, Heidelberg, Germany) equipped with a 63x oil-immersion objective was used. Consecutive thin (about 0.1 µm) sections from the same resin blocks were stained with uranyl-lead or the immunogold procedure using antibodies against proteasome 20S, 19S or 20Sß5i (immunoproteasome; rabbit polyclonal, Calbiochem) subunits, polyubiquitinated proteins, p62 protein, cathepsin D (rabbit polyclonal, H-75, Santa Cruz), CagA (rabbit polyclonal, Austral Biological, San Ramon, CA), VacA (rabbit polyclonal, Austral Biological), urease (UreA, mouse monoclonal, Austral Biological), *H. pylori* outer membrane proteins (OMPs, rabbit polyclonal, Biomeda, Forster City, CA) and NOD1 (rabbit polyclonal, Abnova, Taipei City, Taiwan), followed by anti-mouse or anti-rabbit IgG labelled with 10, 15 or 20 nm gold particles (British Biocell International, Cardiff, UK) and uranyl-lead contrast [1]. Ultrastructural specimens were analyzed by transmission electron microscopy (TEM; Jeol Jem 1200EXIII microscope equipped with Olympus CCD camera Mega View III).

For correlative confocal and electron microscopy (CCEM) the two faces of a thin resin section collected on a 200 mesh gilder finder grid (Electron Microscopy Sciences, Hatfield, PA) were processed separately: one face was first immunostained and viewed by confocal microscopy, as above, then the reverse face of the section was processed for immunogold labelling and observed by TEM after uranyl-lead contrast. The resulting confocal and TEM images of the same area were then overlapped using AdobePhotoshop.

Tests to evaluate the specificity of immunogold labelling were carried out using antibodies absorbed with excess antigen and omitting or substituting the specific antibodies in the first layer of the immunogold procedure. Positive and negative controls were obtained by parallel investigation of specimens already assessed in previous studies [1], [5], [6].

## Results

**Figure 1:**
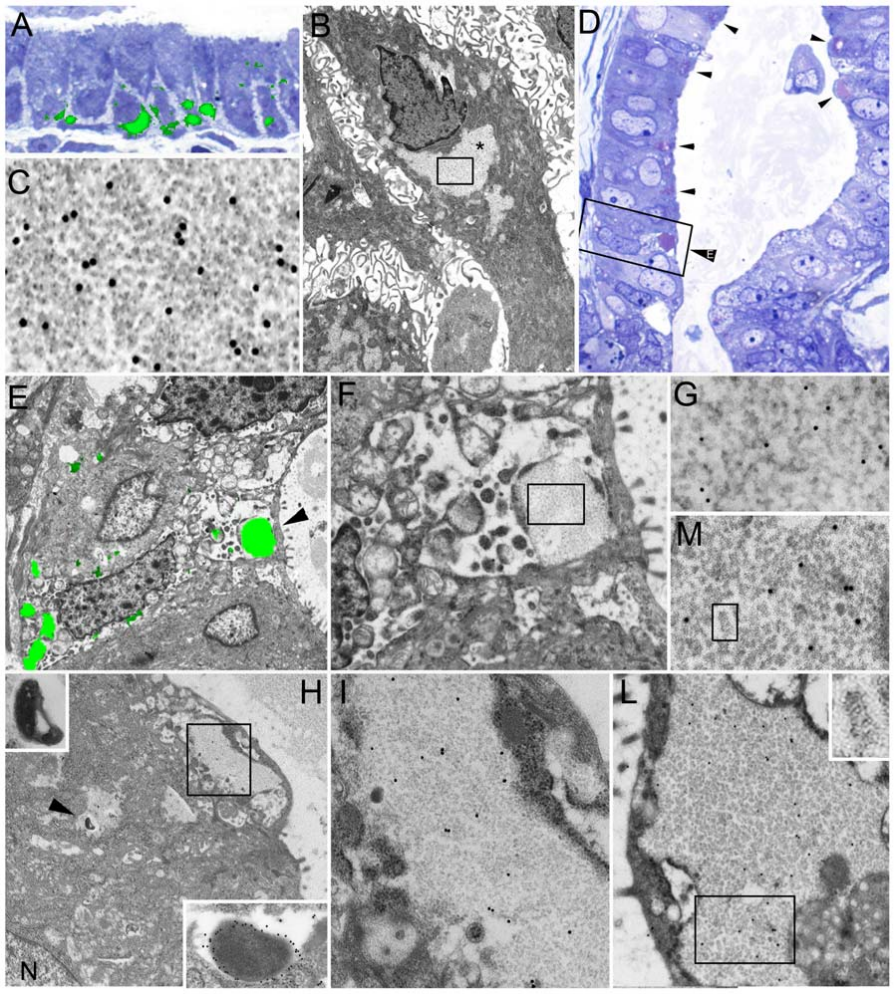
*H. pylori*-colonized non-neoplastic human gastric epithelium (A–C) and well differentiated, gland-forming, deeply invasive, gastric cancer (D–M). (**A**) Foveolar epithelium showing 20S proteasome immunofluorescence (green) of infranuclear PaCSs. Overlapping of confocal fluorescence microscopy and toluidine blue images of a semithin resin section from an aldehyde-osmium-fixed block (500x). (**B**) Juxtanuclear PaCS (asterisk) in a foveolar cell (5,000x) from an adjacent section to **A**. (**C**) High resolution (X 65,000) TEM of a boxed PaCS area in ***B*** to show its particulate ultrastructure and 20S proteasome immunogold reactivity. (**D**) Toluidine blue stained semithin resin section (500x); note pink stained PaCSs (arrowheads), many of which subluminal. (**E**) Correlative confocal and electron microscopy (CCEM), of a consecutive thin section of the boxed area in *D,* shows 20S proteasome immunofluorescence (green spots) on a TEM background (5,000x). The juxtaluminal PaCS (arrowhead) is enlarged in **F** (20,000x) whose boxed area is further enlarged in **G** (50,000x) to illustrate its particulate ultrastructure with 20S proteasome immunogold reactivity. The same proteasome antibody was used on each face of the gilder finder grid used for CCEM. Note several basally located PaCSs in **E**. (**H**) Another thin section (5,000x) from the same tumour block with several supranuclear PaCSs, one of which (arrowhead) is enlarged in the top link inset (18,000x) to show an inner bacterial body (for comparison, another intracellular bacterium positive for H. pylori OMPs immunogold (21,000x) is shown in the bottom right inset), while an juxtaluminal PaCS (boxed area) is enlarged in ***I*** (25,000x) to illustrate NOD1 immunogold reactivity. N, nucleus. (**L**) TEM (20.000x) of another PaCS in another section from the same block immunostained with *H. pylori* OMP antibodies; boxed area enlarged in M (50,000x) to better magnify the immunogold particles and barrel-like putative proteasome particles, one of which (boxed in **M**) is further enlarged in the **L** inset (200,000x) to illustrate the characteristic punctuate-aligned structure.

**Figure 2:**
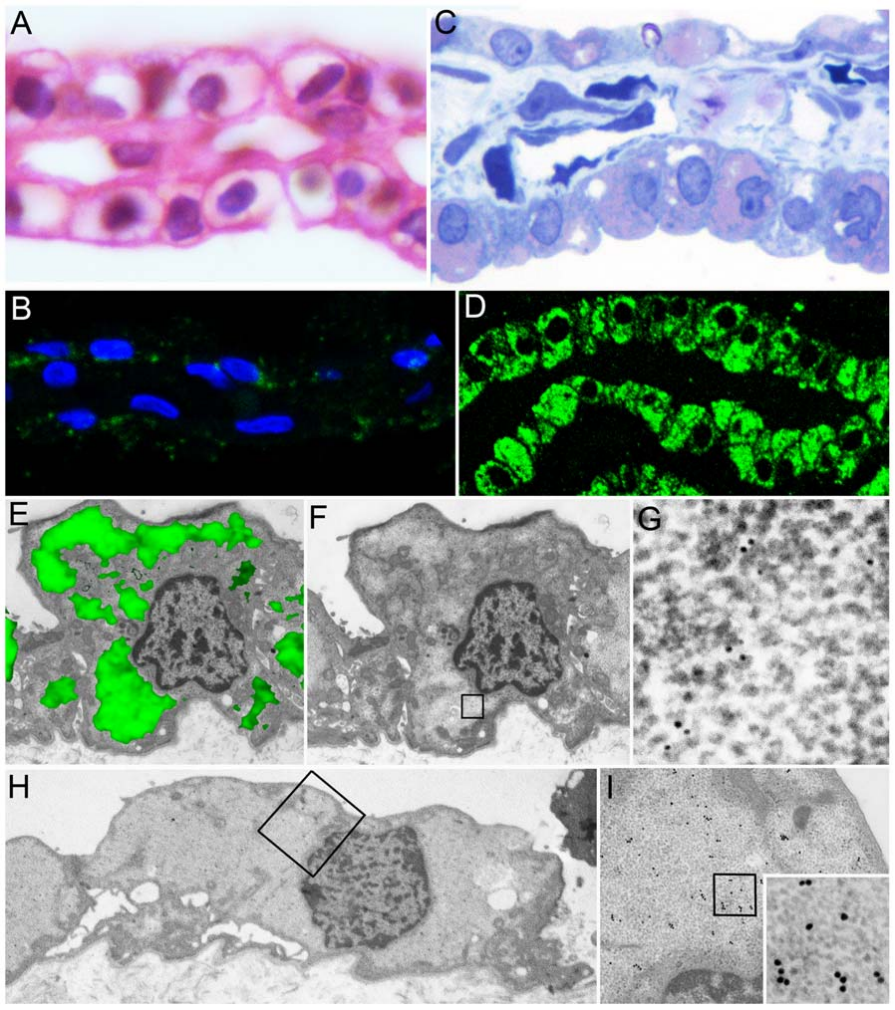
Pancreatic serous microcystic adenoma. (**A**, **B**) Formalin-fixed paraffin sections; note the clear, apparently “empty” cytoplasm of most cells in **A** (hematoxylin-eosin, 1,000x) and the poor reactivity in **B** to proteasome immunofluorescence under confocal microscopy (blue: nuclei; green: proteasome; 800x). (**C**, **D**) Semithin aldehyde-osmium fixed resin sections from the same tumour as in **A** and **B**, show abundant cytoplasmic PaCSs metachromatically stained pink with toluidine blue (**C**, 1,000x) and extensively proteasome immunofluorescent under confocal microscopy (**D**, green: proteasome; 500x). (**E**) CCEM showing proteasome immunofluorescent PaCS (5,000x); the same specimen under TEM only (**F**, 5,000x), boxed area enlarged in **G** (50,000x) shows the PaCS particulate pattern with 20S proteasome immunogold reactivity. (**H, I**) TEM of another specimen of the same tumour in **H** (5,000x), boxed area enlarged in **I** (30,000x) and further enlarged in the inset (50,000x) to show PaCS particulate ultrastructure and 19S proteasome immunogold.

**Figure 3:**
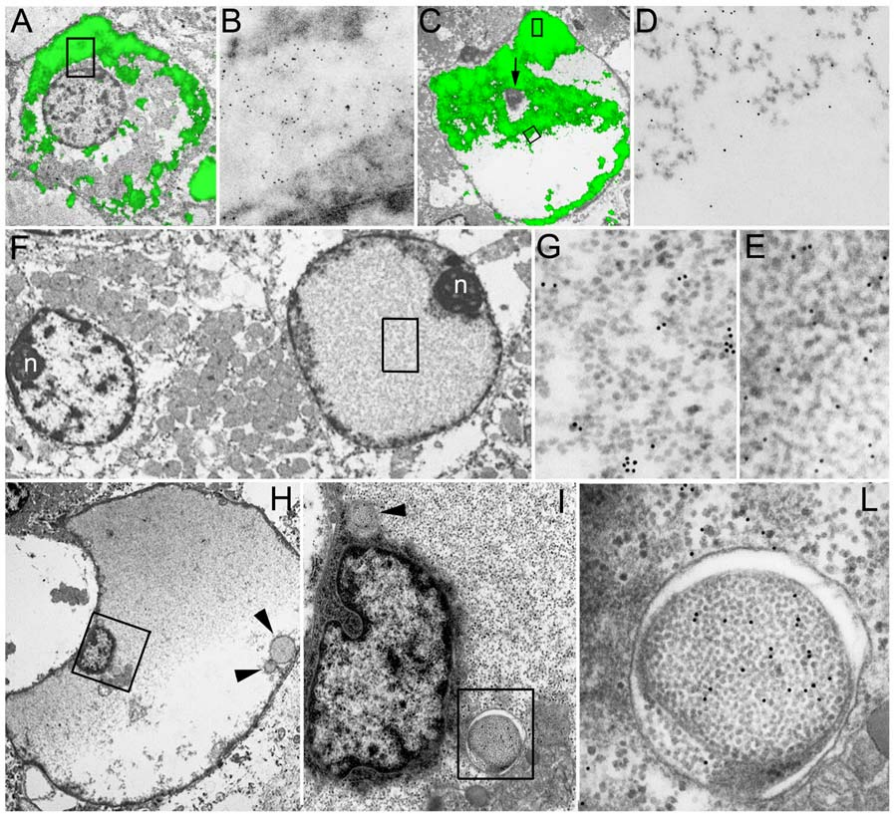
Parotid gland cancer. (**A**) CCEM shows proteasome immunofluorescence (green) distributed inside cytoplasmic PaCSs while sparing the nucleus (**A**, 3,000x). The boxed area is enlarged in **B** (50,000x) to magnify parallel 20S immunogold reactivity despite loss of density of barrel-like particles. Note in **A** mitochondria and clear unreactive areas merging with PaCS areas. In **C** (3,000x) a ballooning tumour cell with remnant of a central picnotic nucleus (arrow) is almost entirely formed by a degenerating PaCS only partly reactive to proteasome fluorescence. The boxed area, taken in **C** at the transition border between its highly and poorly fluorescent parts, shows in **D** (50,000x) the disappearance of particles and 20S proteasome immunogold below the border, in parallel with immunofluorescence. Another boxed area taken from the top of the heavily fluorescent part in *C*, shows well-preserved particles and proteasome immunogold in **E** (50,000x). (**F** and **G**) TEM (5,000x) showing two neoplastic cells, one with well-preserved normal nucleus (left), the other (right) with intranuclear accumulation of PaCS-like particles and proteasome immunogold, part of which is boxed in **F** and enlarged in **G** (50,000x). Note in **F** abundant mitochondria. n, nucleolus. (**H**) Bulging neoplastic cell with a small nucleus surrounded by a rim of dense cytoplasm and immersed in a lake of PaCS-type partly proteasome-reactive, particulate material (4,000x); the boxed area is enlarged in **I** (20,000x) with islets of residual cytoplasm, one of which is further enlarged in **L** (75,000x) to show a qualitative autophagic vesicle with double membrane enclosing a collection of well-preserved immunoproteasome-reactive PaCS-type particles. Note in **H** and **I** (arrowheads) three other double membrane delimited bodies enclosing PaCS-type particles.

**Figure 4:**
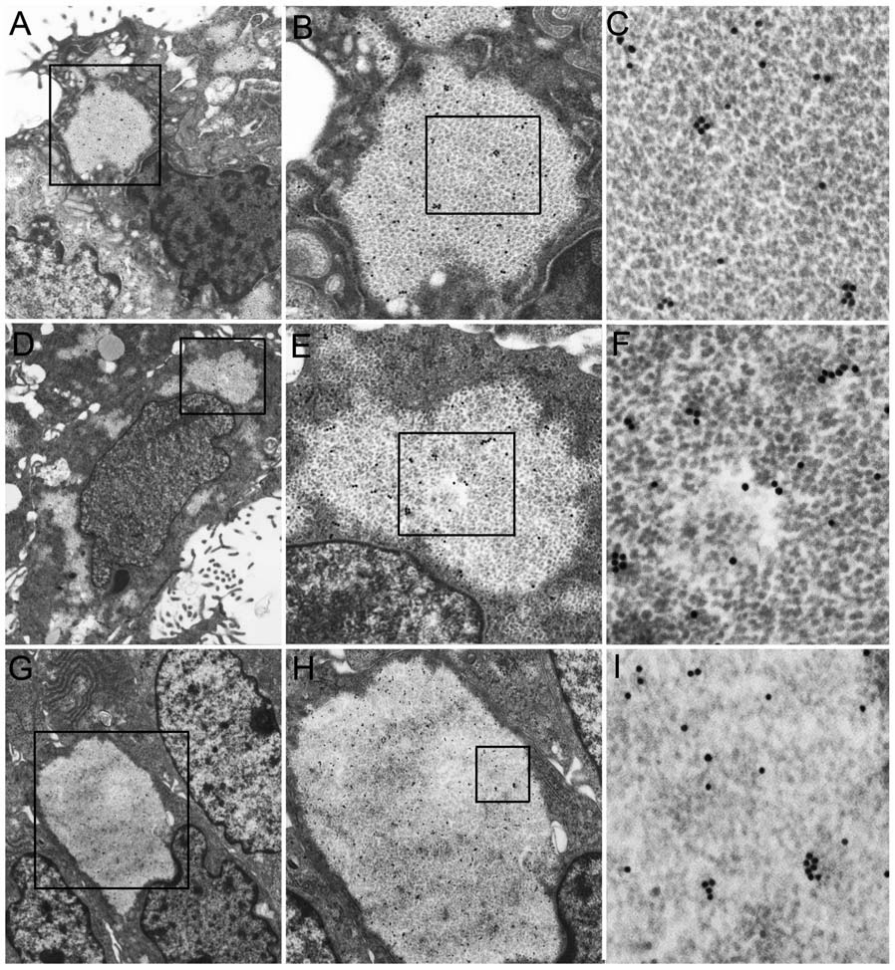
Ovarian serous papillary, cervical endometrioid, and thyroid papillary carcinomas. Juxtanuclear PaCSs in ovarian serous papillary (**A**, 10,000x), cervical endometrioid (**D**, 10,000x), and thyroid papillary carcinoma (**G**, 10,000x), enlarged in **B**, **E**, and **H** (all 25,000x) and even further in **C**, **F**, and **I** (all, 50,000x), respectively, to show their particulate ultrastructure (partially lost in **I** and, focally, in **F**) and 20S proteasome immunogold reactivity.

**Figure 5:**
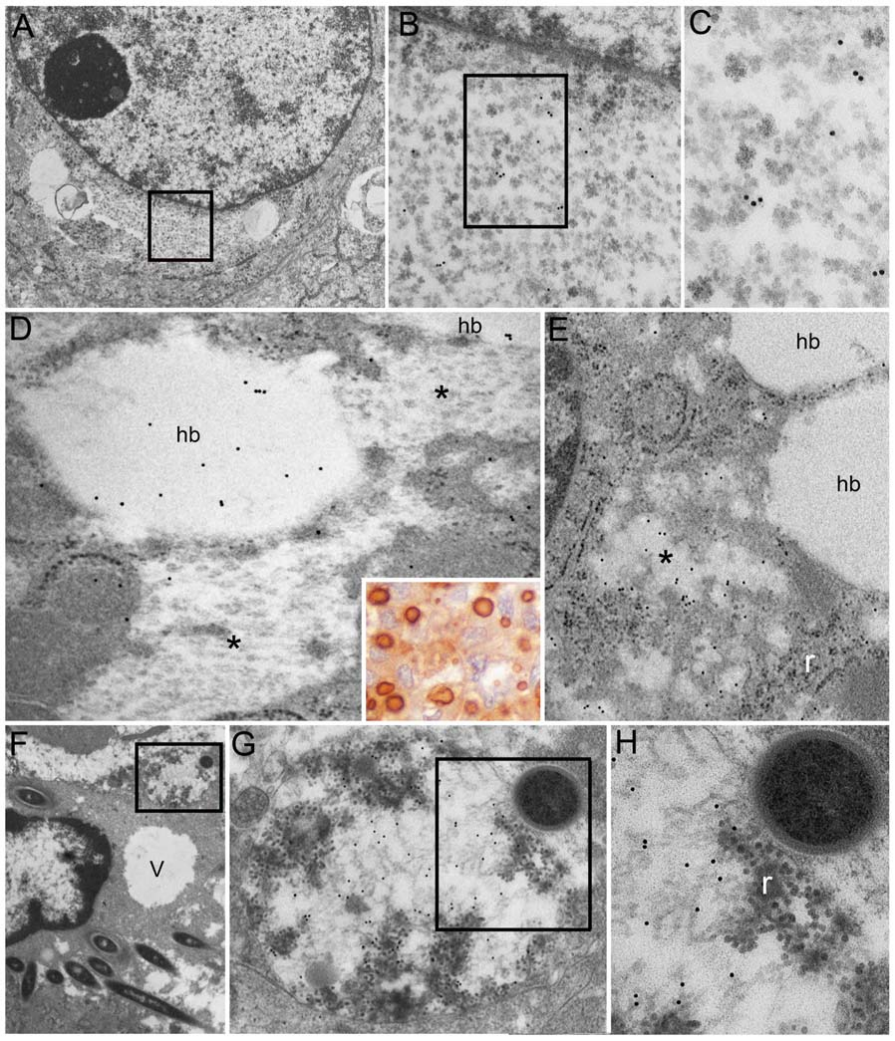
Lung large cell carcinoma, hepatocarcinoma and colon adenocarcinoma. (**A**–**C**) Juxtanuclear PaCS in a lung large cell carcinoma (**A**, 10,000x), boxed area enlarged in **B** (50,000x) whose boxed area is enlarged in **C** (75,000x) to show its particulate structure and proteasome immunogold reactivity. (**D** and **E**) Hepatocellular carcinoma showing both hyaline bodies (hb), reactive for p62 protein in **D** (30,000x) and unreactive for 19S proteasome in **E** (25,000x), and PaCS (asterisks) unreactive for p62 and reactive for 19S proteasome. r, ribosomes. Inset to **D** (450x): hyaline and Mallory bodies react for p62 protein immunoperoxidase in formalin-fixed paraffin sections. (**F**–**H**) Colon adenocarcinoma (**F**, 10,000x) with partly intracellular and partly interstitial bacteria, a cytoplasmic vacuole (v) and a PaCS (boxed) bordering the cell plasma membrane, enlarged in **G** (40,000x) and **H** (75,000x). Note partial lysis of proteasome particles, though retaining 19S proteasome reactivity, development of irregular dense bodies and an encircling peripheral membrane, suggesting transition from a PaCS to an autophagic vacuole, also enclosing ribosome-like particles (r) and a bacterium (top right in **G** and **H**) with capsulated wall typical of Gram-positive organisms.

In accordance with previous studies [1], PaCSs were identified in aldehyde-osmium fixed, resin embedded specimens of *H. pylori*-infected gastric foveolar epithelium as well-defined cytoplasmic areas metachromatically stained pink by toluidine blue and selectively immunofluorescent with antibodies directed against *H. pylori* proteins, proteasome (Figure 1A) or polyubiquitinated proteins. Under electron microscopy, PaCSs showed characteristic barrel-like particles, about 13 nm thick and 15 to 40 nm long, with an inner punctate substructure, and selective immunogold reactivity for proteasome (Figure 1B and C), polyubiquitinated proteins, NOD1, VacA, CagA, urease and OMPs, but not for p62 protein or the lysosomal marker cathepsin D. Using the same procedures, PaCSs were also identified in two of five gastric gland-forming (cohesive, so-called ‘intestinal’) cancers, with all the structural and cytochemical patterns shown in non-tumour gastric epithelium (Figure 1D–M). In addition, cytoplasmic areas reproducing PaCS ultrastructure and proteasome as well as ubiquitinated proteins reactivity, though without *H. pylori* products and NOD1, were detected in pancreatic serous microcystic adenoma (Figure 2), basal cell parotid gland cancer (Figure 3), ovarian serous papillary, uterine cervical and thyroid papillary carcinoma (Figure 4), pulmonary large cell, bronchioloalveolar and hepatocellular carcinoma (Figure 5A–E), apparently in the absence of actual infection. On the other hand, both PaCSs and capsulated (Gram-positive) bacteria were detected inside one case of colon carcinoma (Figure 5F–H). PaCSs were not observed in gastric diffuse, ovarian mucinous and breast ductal cancers. Nuclear areas filled with proteasome-like particles, ultrastructurally and cytochemically indistinguishable from those of PaCS, were also found in one case of parotid cancer (Figure 3F and G).

Compared with those of non-neoplastic cells, tumour PaCSs were more often multifocal, less polarized to the infranuclear cytoplasm, less sharply demarcated from the surrounding cytoplasm and less closely related to ribosome-rich RER. However, large quantitative differences were found from neoplasm to neoplasm, ranging from extensive, fairly uniform areas expanding to occupy most of the cytoplasm in the vast majority of tumour cells, as in pancreatic microcystic adenoma (Figure 2C and D), or in a minority of cells, as in gastric, salivary gland or thyroid cancer, to individual, minute foci inside a few tumour cells as in most other tumours.

In contrast to sequestosomes [14] or autophagosomes [15], both neoplastic and non-neoplastic cell PaCSs retained direct continuity with the surrounding cytoplasm, without interposition of membranes, and lacked p62 protein or cathepsin D immunoreactivity. Of special interest was the p62 protein reactivity of hyaline bodies in hepatocellular carcinoma, in contrast with the unreactivity of most PaCSs observed in the same sections (Figure 5D and E). However, in parotid gland cancer, small, double membrane delimited bodies filled with well-preserved proteasome particles were seen, occasionally, inside PaCSs undergoing partial dissolution (Figure 3H–L). Indeed, TEM detected focal to extensive loss of particles inside PaCSs of some neoplasms, leaving a loose amorphous to filamentous or floccular material often with reduction or even disappearance of proteasome immunoreactivity (Figures 3C–E, 4G–I, and 5H). This lytic process is likely to be involved in the genesis of some amorphous structures or clear areas void of proteasome particles (Figure 3C and D), or of membrane-delimited vacuoles with variable, often irregular content more suggestive of autophagic vacuoles, sometimes with residual particles and proteasome immunoreactivity (Figure 5F–H). No apparent difference of mitochondria ultrastructure was seen in PaCS-positive compared to PaCS-negative cells (not shown).

Proteasome particles were not preserved in gastric biopsies fixed with aldehyde solutions in the absence of osmium post-fixation, embedded in Epon-Araldite or hydrophilic LWR resin, and viewed by TEM, although proteasome protein reactivity was retained, at least in part, thus allowing PaCS recognition. This highlights the crucial fixation conditions required for particle structure preservation and its tendency to dissolve, releasing component proteins, in suboptimal conditions. This may help explain why we failed to detect PaCSs in ordinary formalin-fixed paraffin sections, stained with toluidine blue or proteasome antibodies, even in tumours like pancreatic serous microcystic adenoma where PACSs were extensively seen in aldehyde-osmium resin sections (Figure 2A–D). Thus, poor PaCS fixation by routine acqueous aldehyde solutions may contribute to the extensively “cleared” cytoplasm of serous microcystic adenoma, usually attributed to dissolution of their glycogen content in aqueous fixatives. Both hyaline and Mallory bodies were heavily immunoreactive for p62 protein in formalin-fixed paraffin sections of hepatocellular carcinoma (Figure 5D inset), whereas under the same technical conditions no p62 reactivity was detected in the large clear PaCS-enriched cytoplasmic areas of serous microcystic adenoma.

## Discussion

This investigation documents the presence of PaCSs, the barrel-like particles and proteasome-rich cytoplasmic structures we recently identified in *H. pylori*-infected gastric epithelium [1], in gastric cancer cells. Colocalization inside cancer cell PaCS of *H. pylori* virulence factors like VacA, CagA, urease or outer membrane proteins, the *H. pylori* receptor NOD1, polyubiquitinated proteins, and, occasionally, of *H. pylori* itself, nicely reproduce the ultrastructural and cytochemical patterns of such structures as already detailed in non-neoplastic gastric epithelium and in epithelial cell lines incubated with *H. pylori* culture filtrate. The present findings, together with previous detection of *H. pylori* inside dysplastic intramucosal lesions and invasive cancer [6], support the hypothesis that *H. pylori* has a direct role in the genesis of PaCS and, possibly, in gastric carcinogenesis [3].

Nevertheless, PaCSs with proteasome-rich particles and polyubiquitinated proteins, albeit in the absence of *H. pylori* products and NOD1, were also observed in various epithelial neoplasms arising in the digestive (pancreas, parotid, colon, and liver), respiratory (lung) and female genital tract (ovary, cervix) or even in endocrine glands (thyroid). In addition, proteasome particle-rich structures have also been detected in the nucleus, a known site of proteasome distribution [7], of a parotid gland cancer. The negative findings so far obtained in cases of gastric diffuse, ovarian mucinous and breast ductal carcinomas warrant more extensive investigation. However, the present evidence suggests preferential involvement of neoplasms with serous (proteic) secretory potential and variable degrees of glandular differentiation or histologic grade, from benign (pancreatic serous microcystic adenoma) to highly malignant (pulmonary large cell carcinoma).

In essence, PaCSs may result from focal accumulation inside RER areas of barrel-like particles, likely to be identified with the proteasome functional units, assembled there from their constitutive proteins synthesized at ribosome level (or redistributed there) under cell stimulation, a sort of focal proteasome hyperplasia [1]. As to the nature of the stimulus for this cellular change, previously observed PaCS development in HeLa cells incubated *in vitro* with *H. pylori* culture filtrates and PaCS localization of *H. pylori* itself and its virulence factors in infected gastric epithelium [1], here confirmed and extended to gastric cancer, suggests that *H. pylori* infection is involved in eliciting proteasomal stimulation. This conclusion would fit in with the known role of the ubiquitin-proteasome system (UPS), with special reference to immunoproteasome [8], in mounting a class I MHC-dependent cellular immune response to microbial infection and with our detection of immunoproteasome components inside the PaCS. However, no sign of actual microbial infection has so far been detected in the other PaCS-positive neoplasms investigated, with the only exception of a colon cancer. Thus, besides infection, a more general mechanism eliciting UPS-based cellular responses is likely to be involved. In this context, a link with inflammation and inflammatory cytokines or LPS-induced oxidative stress resulting in neosynthesis of oxidant-damaged misfolded proteins undergoing rapid polyubiquitination seems attractive [9]. It also seems of interest that *H. pylori* and its CagA toxin can induce reactive oxygen species (ROS) in gastric epithelial cells, both directly and secondary to associated gastritis [10], a process which may have a role in carcinogenesis in addition to PaCS formation.

PaCSs clearly differ ultrastructurally and cytochemically from a number of cytoplasmic inclusion bodies, including hepatocellular hyaline or Mallory bodies, inclusions of various neurodegenerative diseases and other sequestosomes or aggresomes [11]–[13], which may share with PaCS a reactivity for polyubiquitinated proteins while usually lacking substantial proteasome reactivity and showing, in addition, p62 protein. Per se, most PaCSs also differ sharply from ubiquitinated protein-reactive, but generally proteasome-negative, autophagic structures, either autophagosomes or autolysosomes, as they lack distinctive features like isolation membranes, p62 protein and inner remnants of cytoplasmic organules or irregular, lysosome-like dense bodies [14]. However, ultrastructural patterns suggesting transition, through progressive loss of particles and proteasome immunoreactivity, from PaCS to amorphous-filamentous structures of the type identified by Bjorkoy et al. [15] as p62-positive sequestosomes destined to autophagy, have been observed in some neoplastic cells, either *in vivo* from tumour samples (this study) or in cultured cell lines [1]. In addition, a few autophagosome-like, double membrane-enveloped bodies filled with proteasome particles were found in parotid gland cancer, inside degenerating PaCSs undergoing dissolution. Thus, although PaCSs and their nuclear equivalents are, per se, clearly different from known inclusion bodies and apparently unrelated to autophagy, when undergoing particle dissolution and proteasome loss (a frequent finding in some neoplasms) they might well be involved in sequestosome formation and autophagy-related degradative processes.

To our knowledge, no specific molecular change is shared by the full spectrum of PaCS-positive cells and neoplasms we have observed, with the possible exception of MAPK-ERK pathway activation [16], [17], either through EGFR overexpression/activation, as in *H. pylori*-infected gastric cells [18] and in pancreatic serous cystic adenoma [19], or through constitutive ERK activation, often secondary to EGFR, RAS or BRAF mutation, as found in hepatocellular, lung, thyroid papillary, ovarian and colorectal cancers [20]–[24]. Interestingly, ERK protein immunoreactivity has been detected inside PaCS of *H. pylori*-infected gastric epithelium [1].

In conclusion, PaCSs may develop in neoplastic and non-neoplastic cells which have undergone functional overstimulation of the ubiquitin-proteasome system due to various stimuli, as for instance microbial infections, oxidative stress or excessive polyubiquitinated protein production/accumulation, to which a contribution of proteasome loss or malfunction preventing rapid degradation of ubiquitinated proteins [9], [25] is possibly to be added. While clarification of PaCS role in cellular homeostasis requires further investigation, its frequent occurrence in neoplasms deserves attention, especially in the light of present therapeutic approaches targeting proteasome [4].
